# Robust preimplantation genetic testing of the common *F8* Inv22 pathogenic variant of severe hemophilia A using a highly polymorphic multi-marker panel encompassing the paracentric inversion

**DOI:** 10.1186/s12959-023-00552-w

**Published:** 2023-10-20

**Authors:** Minh Tam Nguyen, Thanh Tung Nguyen, Duy Bac Nguyen, Thi Mai Nguyen, Kim Ngan Nguyen, Van Nhat Minh Ngo, Van Dieu Nguyen, Ngoc Anh Tran, Mulias Lian, Arnold S. C. Tan, Samuel S. Chong, Tien Truong Dang

**Affiliations:** 1 Department of Anatomy, Vietnam Military Medical University, Hanoi, Vietnam; 2 National Institute of Hematology and Blood Transfusion, Hanoi, Vietnam; 3 Preimplantation Genetic Diagnosis Centre, National University Centre for Women and Children, National University Hospital, Singapore, Singapore; 4 Department of Paediatrics, Yong Loo Lin School of Medicine, National University of Singapore, Singapore, 119228 Singapore; 5 Department of Obstetrics and Gynaecology, Yong Loo Lin School of Medicine, National University of Singapore, Singapore, 119228 Singapore; 6 Department of Laboratory Medicine, National University Hospital, Singapore, Singapore

**Keywords:** Preimplantation genetic testing for monogenic disorders (PGT-M), Hemophilia A (HEMA), Short tandem repeats (STRs), Microsatellite markers, *F8* gene

## Abstract

**Background:**

Hemophilia A (HEMA) is an X-linked bleeding disorder caused by reduced/absent coagulation factor VIII expression, as a result of pathogenic variants in the *F8* gene. Preimplantation prevention of HEMA should ideally include direct pathogenic F8 variant detection, complemented by linkage analysis of flanking markers to identify the high-risk *F8* allele. Linkage analysis is particularly indispensable when the pathogenic variant cannot be detected directly or identified. This study evaluated the suitability of a panel of *F8* intragenic and extragenic short tandem repeat markers for standalone linkage-based preimplantation genetic testing for monogenic disorder (PGT-M) of the Inv22 pathogenic variant, an almost 600 kb paracentric inversion responsible for almost half of all severe HEMA globally, for which direct detection is challenging.

**Methods:**

Thirteen markers spanning 1 Mb and encompassing both *F8* and the Inv22 inversion interval were genotyped in 153 unrelated females of Viet Kinh ethnicity.

**Results:**

All individuals were heterozygous for ≥ 1 marker, ~ 90% were heterozygous for ≥ 1 of the five *F8* intragenic markers, and almost 98% were heterozygous for ≥ 1 upstream (telomeric) and ≥ 1 downstream (centromeric) markers. A prospective PGT-M couple at risk of transmitting *F8* Inv22 were fully informative at four marker loci (2 intra-inversion, 1 centromeric, 1 telomeric) and partially informative at another five (2 intra-inversion, 3 centromeric), allowing robust phasing of low- and high-risk haplotypes. In vitro fertilization produced three embryos, all of which clearly inherited the low-risk maternal allele, enabling reliable unaffected diagnoses. A single embryo transfer produced a clinical pregnancy, which was confirmed as unaffected by amniocentesis and long-range PCR, and a healthy baby girl was delivered at term.

**Conclusion:**

Robust and reliable PGT-M of HEMA, including the common *F8* Inv22 pathogenic variant, can be achieved with sufficient informative intragenic and flanking markers.

**Supplementary Information:**

The online version contains supplementary material available at 10.1186/s12959-023-00552-w.

## Introduction

Hemophilia A (HEMA) is an X-linked recessive bleeding disorder caused by pathogenic variants in the *F8* gene located on chromosome Xq28 [1]. It occurs in ~ 1 in 10,000 live male births [2]. *F8* encodes coagulation factor VIII, an essential component in blood coagulation, and defects in *F8* lead to partial or complete absence or inactivity of this critical protein. The most common pathogenic variants are paracentric inversions where one breakpoint occurs in intron 22 or intron 1 of *F8*. These inversions, commonly referred to as Inv22 and Inv1, account for ~ 45% and ~ 2% of severe HEMA cases, respectively [3]. More than 2000 different pathogenic *F8* variants have been identified in the remaining cases, at much lower frequencies [4], thus making screening of pathogenic *F8* variants inefficient and expensive.

Preimplantation genetic testing for monogenic disorders (PGT-M) avoids transmission of monogenic disease in at-risk families by selective transfer of only unaffected embryos to women after in vitro fertilization. Robust and reliable PGT-M should ideally include direct detection of the pathogenic *F8* variant, coupled with indirect linkage analysis of tightly-linked informative markers flanking the pathogenic variant, to track transmission of the high- and low-risk *F8* alleles. We previously developed a single-tube panel of 13 short tandem repeat (STR) markers to simplify informative marker identification for use in linkage-based PGT-M of HEMA to complement direct detection of pathogenic *F8* variants in an at-risk couple [5]. STR markers not only effectively track the pathogenic variant [6] but also serve to detect DNA contamination and allele dropout (ADO), and together with direct pathogenic variant detection, increase diagnostic confidence of PGT-M results [7].

Where the pathogenic *F8* variant cannot be identified or detected directly, however, PGT-M relies entirely on indirect linkage analysis, but at least one affected family member is needed in addition to the at-risk couple, in order to establish disease allele phase of each marker. One example is the common Inv22 pathogenic variant, which involves a paracentric inversion of the interval between a sequence in intron 22 of *F8* and a homologous sequence ~ 575 kb telomeric to it. Molecular diagnosis of this pathogenic variant usually requires long-distance PCR (LD-PCR) or inverse PCR (I-PCR) from large amounts of high molecular weight genomic DNA [8, 9]. Although direct Inv22 detection from whole genome amplified single blastomeres using LD-PCR has been demonstrated in one PGT-M report [10], ADO of the pathogenic allele was observed in one blastomere result, making LD-PCR unsafe as a standalone PGT-M test for Inv22 HEMA. For most laboratories, standalone linkage analysis using informative linked markers is the primary PGT-M option available to carriers of Inv22, which accounts for nearly half of all severe HEMA alleles globally. However, because of the large inversion interval of nearly 600 kb, misdiagnosis or inconclusive results may arise due to meiotic recombination between marker and breakpoint on either end of the inversion interval, especially when insufficient informative and tightly linked markers are available to encompass the inversion interval. In this study, we assessed our 13-marker panel for standalone linkage-based PGT-M of HEMA involving the pathogenic *F8* Inv22 variant.

## Materials and methods

### Population samples

Blood samples were collected from 50 unrelated healthy females and 103 HEMA carriers at the National Institute of Hematology and Blood Transfusion (NIHBT) in Hanoi, Vietnam from September 2018 to June 2019. DNA extraction was performed using the Blood DNA Extraction QIAamp® DNA Mini Kit (Qiagen, Hilden, Germany) following manufacturer’s protocol. Extracted DNAs were genotyped for STR markers to determine markers’ heterozygosity values. This study was approved by the Ethics Council in Biomedical Research of Vietnam Military Medical University. Written informed consent was obtained from all participants.

### Marker genotyping and analysis

Each multiplex PCR was performed in a 20 µl reaction volume consisting of 50–100 ng genomic DNA, 1X Qiagen Multiplex Mastermix (Qiagen), and 0.15–0.70 µM each of the relevant forward and reverse primer (Supplemental Table S1). Thermal cycling involved an initial 15-min enzyme activation at 95 °C, 30 cycles of denaturation at 94 °C for 30 s, annealing at 60 °C for 1 min 30 s, and extension at 72 °C for 1 min, and a final extension at 60 °C for 30 min. A 1 µl aliquot of PCR product was subjected to an extension labeling reaction in a 20 µl mixture, which consisted of 1X Qiagen Multiplex Mastermix and 0.2 µM of 6-Fam-labeled M13-1 primer (5’-GGTTTTCCCAGTCACGAC-3’), Hex-labeled M13-2 primer (5’-GTAAAACGACGGCCAGTG-3’), and Ned-labeled M13-3 primer (5’-CATGGTCATAGCTGTTTCCTG-3’) (Supplemental Table S1). Thermal cycling involved an initial enzyme activation at 95 °C for 15 min, followed by 10 cycles of denaturation at 94 °C for 30 s, annealing at 60 °C for 1 min 30 s, and extension at 72 °C for 1 min, with a final extension at 60 °C for 30 min.

A 1 µl aliquot of fluorescent extension labeling product was mixed with 8.5 µl of Hi-Di Formamide (Applied Biosystems-Thermo Fisher Scientific) and 0.5 µl of GeneScan 500 LIZ dye size standard (Applied Biosystems), denatured at 95 °C for 5 min, cooled to 4 °C, and resolved in an ABI 3130XL Genetic Analyzer (Applied Biosystems). Post-electrophoresis analysis was performed using GeneMapper 5.0 software (Applied Biosystems).

Allele frequency, expected heterozygosity (H_e_), and observed heterozygosity (H_o_) of the 13 microsatellite markers were calculated using Microsoft Excel [11].

### PGT-M of pathogenic F8 Inv22 variant of HEMA

Linkage-based PGT-M of HEMA was performed in a family segregating with the pathogenic *F8* Inv22 variant. The carrier female, her unaffected husband, and an affected son were genotyped at all 13 STR markers to establish the disease haplotype phase in this family. PGT-M was subsequently performed on trophectoderm biopsy samples of three day-5 blastocysts of the couple. Trophectoderm samples were subjected to whole genome amplification (WGA) using the REPLI-g Single Cell Kit (Qiagen) according to manufacturer’s protocol. A 1 µl aliquot of WGA product was subjected to a single-tube multiplex PCR amplification of all 13 STRs and the sex chromosome discriminating *AMELX/Y* marker in a 20 µl reaction consisting of 1X Qiagen Multiplex Mastermix and 0.15–0.7 µM of each primer. Thermal cycling was performed as described above except that 40 cycles were used. Extension labeling was performed as described above. Written informed consent was obtained from the couple.

## Results

### Evaluation of marker heterozygosity and polymorphism

**Fig. 1:**
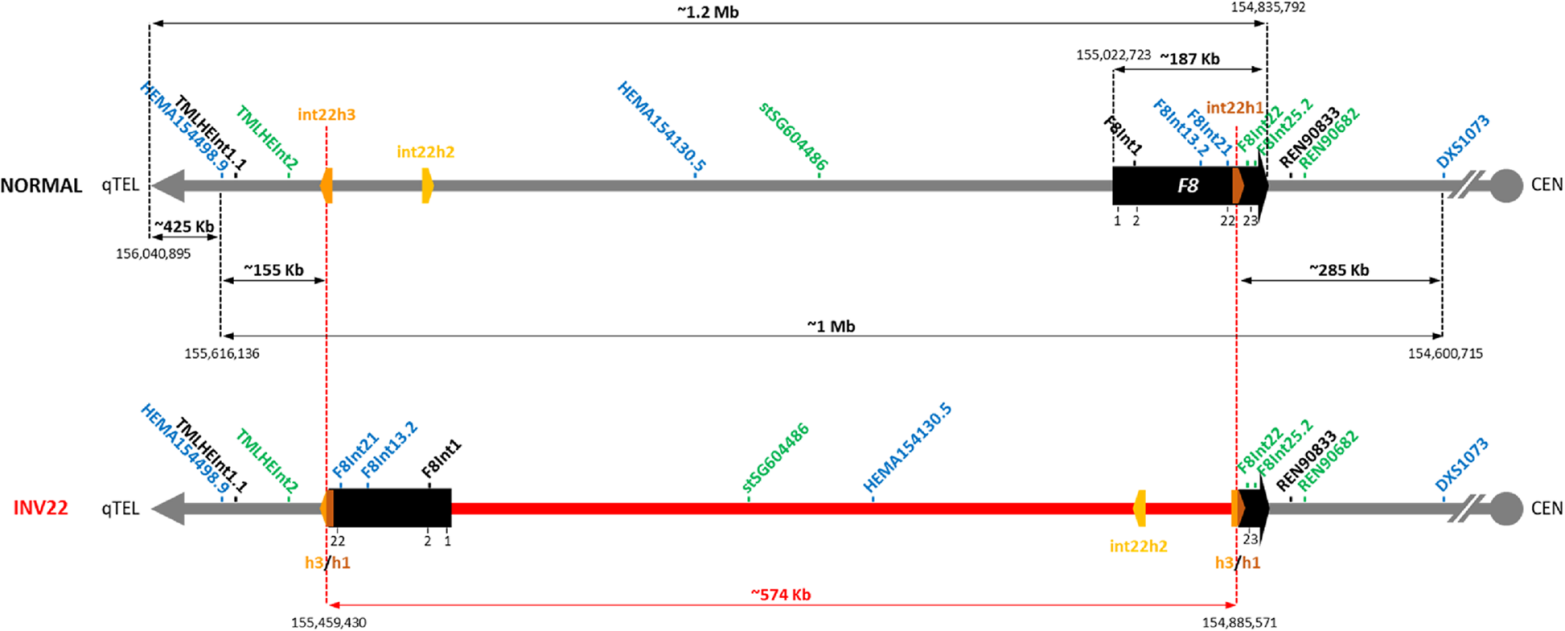
Structure of chromosome X band q28 between *F8* and the telomere. Top line depicts a normal *F8* allele, while bottom line depicts a pathogenic *F8* Inv22 allele. Positions of 13 STR markers spanning and flanking the inversion interval are shown

The single-tube multiplex PCR panel consists of 13 markers, all of which are located within 1 Mb of the *F8* gene with five located upstream, five intragenic, and three downstream of *F8* (Fig. 1). These markers were previously shown to be highly polymorphic in the ethnic Chinese and Caucasian populations [5], and we have now further analyzed 153 unrelated females from the majority Kinh ethnic group in Vietnam. A majority of STRs successfully amplified from all samples except for *F8Int13.2* and *stSG604486*, which failed to amplify in 2 and 6 samples, respectively. A combined total of 226 allele sizes were observed, with 10–28 alleles observed per marker and allele frequencies ranging between 0.0033 and 0.3399 (Supplemental Table S2). Marker H_e_ values ranged from 0.61 (*F8Int25.2*) to 0.90 (*HEMA154130.5* and *HEMA154498.9*), while H_o_ values ranged from 0.58 (*REN90833*) to 0.90 (*REN90682*) (Table 1).

**Table 1:** Observed and expected heterozygosity values of 13 HEMA STR markers in the Vietnamese Kinh compared to the Chinese and Caucasions

**Markers**	**Heterozygous**	**Total**	**Observed heterozygosity (H**_**o**_**)**			**Expected heterozygosity (H**_**e**_**)**		
			**VT**	**CH**^**a**^	**CAU**^**a**^	**VT**	**CH**^**a**^	**CAU**^**a**^
*DXS1073*	127	153	0.83	0.73	0.51	0.86	0.69	0.66
*REN90682*	138	153	0.90	0.68	0.53	0.88	0.7	0.58
*REN90833*	89	153	0.58	0.49	0.43	0.85	0.49	0.43
*F8Int25.2*	116	153	0.76	0.50	0.47	0.61	0.48	0.51
*F8Int22*	112	153	0.73	0.52	0.55	0.76	0.51	0.56
*F8Int21*	111	153	0.73	0.48	0.51	0.81	0.48	0.51
*F8Int13.2*	105	151	0.70	0.63	0.57	0.85	0.58	0.65
*F8Int1*	110	153	0.72	0.45	0.43	0.77	0.48	0.49
*stSG604486*	107	147	0.73	0.59	0.51	0.87	0.54	0.52
*HEMA154130.5*	133	153	0.87	0.77	0.59	0.90	0.75	0.57
*TMLHEInt2*	131	153	0.86	0.63	0.67	0.88	0.60	0.65
*TMLHEInt1.1*	92	153	0.60	0.61	0.57	0.86	0.56	0.53
*HEMA154498.9*	124	153	0.81	0.84	0.78	0.90	0.77	0.79

*VT* Vietnamese Kinh, *CH* Singapore Chinese, *CAU* Coriell Cell Repositories Caucasian Human Variation Panel

^a^From Zhao et al., 2017 [5]

All individuals were heterozygous for at least one marker (Supplemental Figure S1A) and ~ 90% of individuals were heterozygous for at least one of the five *F8* intragenic markers (Supplemental Figure S1B). Furthermore, almost 98% of individuals were heterozygous for at least one upstream (telomeric) and one downstream (centromeric) markers (Supplemental Figure S1C). These results further confirm the high polymorphism of this tridecaplex marker panel and likelihood that it will provide sufficient informative markers, either for standalone linkage-based PGT-M or when used in parallel with direct detection of any pathogenic *F8* variant, including the common Inv22 pathogenic variant.

### Standalone linkage-based PGT-M in a HEMA family at-risk of transmitting F8 Inv22

**Fig. 2:**
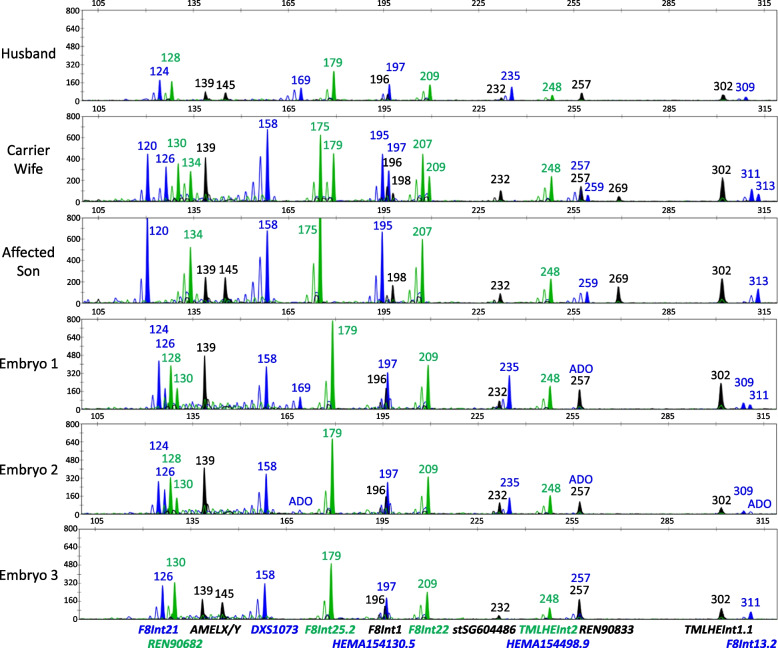
Electropherogram results from the HEMA *F8* Inv22 PGT-M case, after single-tube multiplex-PCR of 13 *F8*-associated STR markers and the sex chromosome discriminating *AMELX/Y* marker. Results of the couple and their affected son were generated from peripheral blood DNA, while the embryo results were generated from trophectoderm biopsies. ADO, allele dropout

**Fig. 3:**
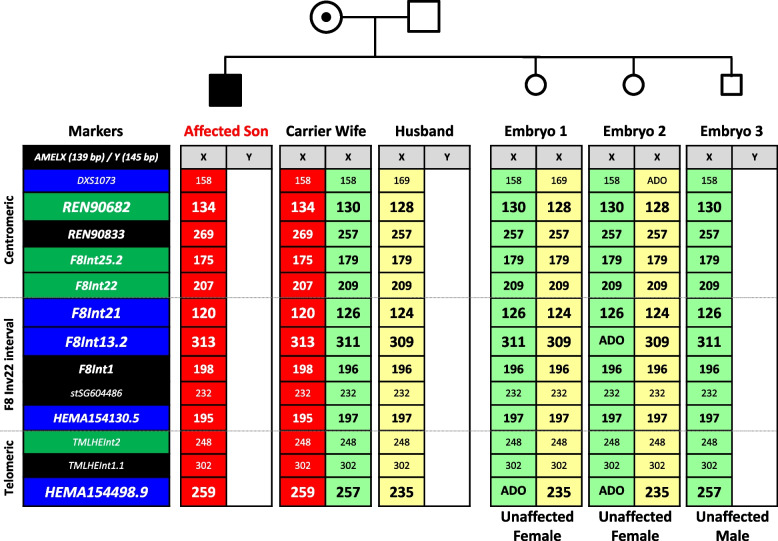
Linked haplotype analysis of the HEMA *F8* Inv22 PGT-M case. The high-risk marker haplotype of the carrier wife (in red) was established from the marker alleles present in the affected son. All three embryos inherited the maternal low-risk haplotype (in green) with no evidence of marker recombination, and are thus unaffected. ADO, allele dropout

The marker panel was applied to PGT-M in a couple at risk of transmitting the intron 22 inversion. Blood samples of a carrier female, her unaffected husband, and an affected son were received after genetic counseling, and genotyped at all 13 STR markers. The couple were fully informative at four marker loci, of which two were within the Inv22 interval (*F8Int13.2*, *F8Int21*), and one each were centromeric (*REN90682*) and telomeric (*HEMA154498.9*). In addition, there were five partially informative markers, of which two were within the Inv22 interval (*F8Int1, HEMA154130.5*) and three were centromeric (*REN90833, F8Int25.2, F8Int22*). These nine fully and partially informative markers were used to establish the disease haplotype phase in the family. The couple subsequently underwent IVF and three embryos were obtained and biopsied on day 5, followed by PGT-M of the biopsied trophectoderm tissues. All three embryos were diagnosed to be unaffected, having inherited the maternal low-risk haplotype (Figs. 2 and 3). A single embryo of the best morphology score (embryo 2) was transferred, leading to a clinical pregnancy. An amniocentesis was performed at the 16^th^ week of gestation, and unaffected status of the fetus was confirmed using long-range PCR [8] (data not shown). A healthy baby girl was delivered at term.

## Discussion

More than 2000 HEMA pathogenic variants have been described in the 186 kb *F8* causative gene, including inversions, deletions, insertions, duplications, and a wide range of point mutations [4]. Prenatal and preimplantation genetic testing remain crucial enabling tools for at-risk families to avoid affected pregnancies. Whenever possible, PGT-M for HEMA should include direct pathogenic variant detection together with linkage analysis of markers flanking the pathogenic variant, to avoid misdiagnosis due to ADO at the pathogenic variant site. Approximately 45% of severe HEMA cases are caused by Inv22, a ~ 575 kb paracentric inversion within chromosome Xq28 that truncates the *F8* gene, mediated by non-allelic homologous recombination between duplicons within intron 22 and distally towards the telomere [1]. Chen et al. successfully demonstrated detection of the Inv22 pathogenic variant in 15 whole genome amplified single lymphocytes of an Inv22 carrier cell line using LD-PCR [10]. When the LD-PCR assay was applied to an actual PGT-M case, complemented with linkage analysis using four informative markers, ADO of the pathogenic variant was detected in one of eight blastomeres. If not for the concurrent analysis of linked markers, this carrier embryo would have been misdiagnosed as normal.

Given the large size of the Inv22 inversion, it is important to ensure that sufficient markers are available within and flanking the inversion breakpoints to ensure that the linkage-based PGT-M assay is robust to amplification failure, allele dropout, and/or unobserved marker-variant recombination. Bui et al. reported using four linked polymorphic STR markers (comprising two telomeric, one intragenic, and one centromeric markers) in 12 linkage-based PGT-M cases for HEMA in Vietnam [12]. However, not all markers were found to be informative in every couple, resulting in some cases where no informative marker was available on one flank of the pathogenic variant, running the risk of misdiagnosis due to unobserved recombination between pathogenic variant and marker on the other flank. In this study, we demonstrate PGT-M of this pathogenic variant hotspot using a 13-marker panel that straddles the almost 600 kb inversion interval. This STR panel, which is highly polymorphic in the Caucasian and Chinese populations [5], has now been shown to be highly polymorphic in a third population group, the majority Kinh ethnic group in Vietnam. In fact, these markers displayed generally higher observed and expected heterozygosity values in the Kinh compared to the Chinese and Caucasian populations (Table 1) and can be used in most if not all at-risk couples regardless of pathogenic *F8* variant.

Although intragenic markers have been recommended as the only option for marker choice for PGT-M of HEMA [13], extragenic markers are suitable alternatives when all intragenic markers are uninformative, so long as they are tightly linked and < 1 Mb away from the pathogenic variant to minimize the likelihood of marker-variant recombination to ≤ 1%. In the case of the Inv22 pathogenic variant, however, informative *F8* intragenic markers alone may be insufficient to exclude meiotic recombination occurring within the ~ 575 kb inversion interval. The highly polymorphic 13-marker panel increases the likelihood of finding informative markers within as well as flanking either end of the inversion, thus maximizing ability to detect any recombination occurring within the inversion interval. Importantly, despite straddling the hotspot inversion’s duplicons, the distance between the panel’s two most distant markers (*HEMA154498.9* and *DXS1073*) is only ~ 1 Mb (Fig. 1), minimizing the likelihood of recombination between any intra-inversion site and flanking marker to ≤ 1%.

In the first HEMA PGT-M case conducted using the single-tube 13-marker PCR panel as a standalone assay to detect the pathogenic *F8* Inv22 variant, four fully informative markers were identified in the couple. They included two markers which were within the Inv22 interval (*F8Int21, F8Int13.2*), one telomeric (*HEMA154498.9*), and one centromeric (*REN90682*). In addition, five markers were partially informative in the couple, of which two were within the inversion interval (*F8Int1, HEMA154130.5*) and three centromeric (*F8Int22, F8Int25.2, REN90833*). Trophectoderm samples from three day-5 embryos were analyzed using these nine markers, and all embryos were unequivocally diagnosed to be unaffected, each having inherited the non-recombinant maternal low-risk allele.

In conclusion, sufficient tightly linked informative markers can be reliably used in PGT-M of HEMA, either as a standalone linkage-based test or to complement pathogenic *F8* variant detection.

### Supplementary Information

**Additional file 1: Supplemental Table S1.** Thirteen STR markers spanning and flanking the F8 Inv22 interval, and the AMELX/Y gender discriminating marker. **Supplemental Table S2.** Observed allele frequencies of 13 STR markers in the Vietnamese Kinh. **Supplemental Figure S1.** Heterozygosities of *F8*‐associated short tandem repeat (STR) markers in the Vietnamese Kinh population. (A) Distribution of individuals heterozygous for different numbers of STRs. (B) Distribution of individuals heterozygous for different numbers of intragenic STRs. (C) Distribution of individuals heterozygous for different numbers of STRs upstream and downstream of the *F8 *gene.

## Data Availability

The data that supports the findings of this study are available in the supplementary material of this article.

## Abbreviations

ADO: Allele dropout
HEMA: Hemophilia A
H_e_: Expected heterozygosity
H_o_: Observed heterozygosity
I-PCR: Inverse PCR
LD-PCR: Long-distance PCR
PGT-M: Preimplantation genetic testing for monogenic disorders
STR: Short tandem repeat
WGA: Whole genome amplification
