# Supplementary material for: Robust preimplantation genetic testing of the common F8 Inv22 pathogenic variant of severe hemophilia A using a highly polymorphic multi-marker panel encompassing the paracentric inversion
Source: Thromb J. 2023 Oct 20;21:108. doi: 10.1186/s12959-023-00552-w (PMC10588207; doi:10.1186/s12959-023-00552-w)
Supplement: Supplementary file 1 — Additional file 1: Supplemental Table S1. Thirteen STR markers spanning and flanking the F8 Inv22 interval, and the AMELX/Y gender discriminating marker. Supplemental Table S2. Observed allele frequencies of 13 STR markers in the Vietnamese Kinh. Supplemental Figure S1. Heterozygosities of F8‐associated short tandem repeat (STR) markers in the Vietnamese Kinh population. (A) Distribution of individuals heterozygous for different numbers of STRs. (B) Distribution of individuals heterozygous for different numbers of intragenic STRs. (C) Distribution of individuals heterozygous for different numbers of STRs upstream and downstream of the F8 gene. [file 12959_2023_552_MOESM1_ESM.pdf]

Supplemental Table S1. Thirteen STR markers spanning and flanking the *F8* Inv22 interval, and the *AMELX/Y* gender discriminating marker.

Marker	Location	Repeat	Primer	Sequence (5'-3')	Conc. (µM)	Size (bp)
DXS1073	Centromeric	$(TG)_n$	X1F	[†]GGTTTTCCCAGTCACGACGAATGCCCTCTCCGAGTTATTAC	0.25	152 – 172
			X1R	GTTTCTGAGATTGGTGGCCTTTGAAAC	0.25	
REN90682	Centromeric	$(AC)_n$	X2F	[‡]GTAAAACGACGGCCAGTGGAAAATTCTCCTCTCCTGCTCC	0.2	129 – 143
			X2R	CCTTCTTTCTTCAAATGATGCTTGG	0.2	
REN90833	Centromeric	$(TTAT)_n$	X3F	[§]CATGGTCATAGCTGTTTCCTGGTCAATATGTCTTGGTCTGGC	0.4	251 – 275
			X3R	GTTTCTCCCATTCTATTCCTAAATAAGATAGC	0.4	
F8Int25.2	Centromeric	$(TG)_n$	X4F	[‡]GTAAAACGACGGCCAGTGCTTGAGACTTGGCAGACTCC	0.2	175 – 181
			X4R	GTTTCCTTTAGAATCACTCTTGGTGTG	0.2	
F8Int22	Centromeric	$(GT)_n$	X5F	[‡]GTAAAACGACGGCCAGTGGAATGCACAGCCTATCCTCC	0.2	207 – 217
			X5R	GTCAAGGTGTCAAATCCCACG	0.2	
F8Int21	Intra-inversion	$(AC)_n$	X6F	[†]GGTTTTCCCAGTCACGACCCACTACCAAATCATTGAGCC	0.2	116 – 128
			X6R	GGTCCATTAGCTTATGTGAGTC	0.2	
F8Int13.2	Intra-inversion	$(AC)_n$	X7F	[†]GGTTTTCCCAGTCACGACTCAACAAAACTGAACAACTAGAAGG	0.7	309 – 321
			X7R	AATCTGGATGGCTTCAAGCTC	0.7	
F8Int1	Intra-inversion	$(AC)_n$	X8F	[§]CATGGTCATAGCTGTTTCCTGGACCCTGTACTTTTACCATTGGC	0.2	188 – 198
			X8R	GTTTCTCAGCAACTCGACTTCTGGC	0.2	
stSG604486	Intra-inversion	$(AC)_n$	X9F	[§]CATGGTCATAGCTGTTTCCTGGGCAATTTCAGATCACTTCTTTC	0.6	226 – 238
			X9R	GTTTGTTACGGTTTTCTTTGTGGC	0.6	
HEMA154130.5	Intra-inversion	$(CT)_n$	X10F	[†]GGTTTTCCCAGTCACGACAATCTATGTGAGTCAGCCCC	0.4	179 – 215
			X10R	GTTTCTCAAGTAGAAAAGTCCTGTGGC	0.4	
TMLHEInt2	Telomeric	$(GT)_n$	X11F	[‡]GTAAAACGACGGCCAGTGCTGTCTGATACAACTGCTGATAC	0.25	234 – 256
			X11R	GGGTTAGTGGTACTCAAACAC	0.25	
TMLHEInt1.1	Telomeric	$(GAAA)_n$	X12F	[§]CATGGTCATAGCTGTTTCCTGGCTGCAACTGTGTCACTG	0.3	289 – 319
			X12R	GTTTCCCTACTGTCTTGACTATTGTGGC	0.3	
HEMA154498.9	Telomeric	$(TC)_n$	X13F	[†]GGTTTTCCCAGTCACGACGGATTCCAGTGACATTGACTCTATC	0.3	235 – 267
			X13R	GTATCTACTGTTACATGGACTTGGG	0.3	
AMELX/Y	-	-	X14F	[§]CATGGTCATAGCTGTTTCCTGCTGGGCTCTGTAAAGAATAGTG	0.15	X: 139<br>Y: 145
			X14R	GTTTCTCAACCATCAGAGCTTAAACTGG	0.15	

Underlined sequences of forward primers are M13-1 (†), M13-2 (‡), and M13-3 (§) tails. Double-underlined sequences of reverse primers are added nucleotides.

Supplemental Table S2. Observed allele frequencies of 13 STR markers in the Vietnamese Kinh.

F8Int21		***DXS1073***		***HEMA154130.5***		***D11S2362***		***F8Int13.2***	
**Allele**	**Frequency**	**Allele**	**Frequency**	**Allele**	**Frequency**	**Allele**	**Frequency**	**Allele**	**Frequency**
114	0.0163	142	0.0033	168	0.0033	232	0.0033	304	0.0033
117	0.0033	144	0.0065	189	0.0033	234	0.0915	307	0.0132
119	0.0196	151	0.0131	190	0.0065	235	0.0359	308	0.0232
120	0.1242	152	0.0033	191	0.0098	236	0.2418	309	0.1126
121	0.1961	153	0.0098	192	0.0261	237	0.0261	310	0.0960
122	0.0523	154	0.0392	193	0.0359	238	0.0915	311	0.1854
123	0.0392	155	0.0556	194	0.0719	239	0.0294	312	0.1623
124	0.2484	156	0.2614	195	0.0621	240	0.0163	313	0.2417
125	0.2712	157	0.0261	196	0.1536	241	0.0033	314	0.0298
126	0.0131	158	0.1961	197	0.1895	246	0.0033	315	0.0993
127	0.0098	159	0.1471	198	0.0327	250	0.0033	316	0.0099
133	0.0065	160	0.0261	199	0.0033	252	0.0033	317	0.0199
		161	0.0490	200	0.0065	253	0.0098	319	0.0033
		162	0.0033	203	0.0033	254	0.0229		
		163	0.0065	204	0.0163	255	0.0229		
		164	0.0033	206	0.0425	256	0.0490		
		165	0.0163	207	0.0131	257	0.0327		
		166	0.0098	208	0.1373	258	0.0915		
		167	0.0131	209	0.0359	259	0.0588		
		168	0.0261	210	0.0882	260	0.0490		
		169	0.0523	211	0.0294	261	0.0458		
		170	0.0098	212	0.0163	262	0.0196		
		171	0.0196	213	0.0033	263	0.0229		
		178	0.0033	214	0.0033	264	0.0065		
				215	0.0033	265	0.0098		
				217	0.0033	266	0.0033		
						267	0.0033		
						273	0.0033		

REN90682		*F8Int25.2*		*F8Int22*		*TMLHEInt2*		*F8Int1*	
**Allele**	**Frequency**	**Allele**	**Frequency**	**Allele**	**Frequency**	**Allele**	**Frequency**	**Allele**	**Frequency**
121	0.0033	161	0.0033	196	0.0033	233	0.0065	192	0.0098
123	0.0033	173	0.0065	206	0.0065	234	0.0163	193	0.0098
124	0.0196	174	0.0523	207	0.1111	235	0.0033	194	0.0490
125	0.0098	175	0.1144	208	0.2908	236	0.0065	195	0.0261
126	0.0458	176	0.1111	209	0.1503	237	0.0131	196	0.3137
127	0.0784	177	0.1307	210	0.3399	239	0.0033	197	0.2255
128	0.1699	178	0.1176	211	0.0458	243	0.0033	198	0.2582
129	0.2222	179	0.2418	212	0.0425	244	0.0065	199	0.0752
130	0.0294	180	0.2092	213	0.0065	245	0.0098	200	0.0065
131	0.0196	181	0.0033	220	0.0033	246	0.0033	201	0.0065
132	0.0196	191	0.0098			247	0.1013	202	0.0065
133	0.0523					248	0.0882	205	0.0033
134	0.1176					249	0.2288	207	0.0033
135	0.1176					250	0.0523	208	0.0065
136	0.0098					251	0.1438		
138	0.0065					252	0.0621		
139	0.0752					253	0.1078		
						254	0.0229		
						255	0.0392		
						256	0.0033		
						257	0.0523		
						258	0.0163		
						260	0.0065		
						261	0.0033		

stSG604486		*REN90833*		*TMLHEInt1.1*	
**Allele**	**Frequency**	**Allele**	**Frequency**	**Allele**	**Frequency**
231	0.0612	252	0.0033	297	0.0098
232	0.1122	256	0.0033	298	0.0131
233	0.1735	257	0.2582	299	0.0033
234	0.0442	258	0.1340	301	0.1895
235	0.2007	260	0.1895	302	0.1438
236	0.0918	261	0.0359	303	0.1895
237	0.1293	262	0.0098	305	0.0980
238	0.0136	264	0.0065	306	0.1144
239	0.1054	265	0.0229	307	0.1471
240	0.0306	266	0.0163	309	0.0196
241	0.0272	267	0.0131	310	0.0261
245	0.0034	268	0.0229	311	0.0392
257	0.0068	269	0.0784	312	0.0033
		270	0.1111	315	0.0033
		271	0.0523		
		272	0.0033		
		273	0.0131		
		274	0.0163		
		276	0.0065		
		279	0.0033		

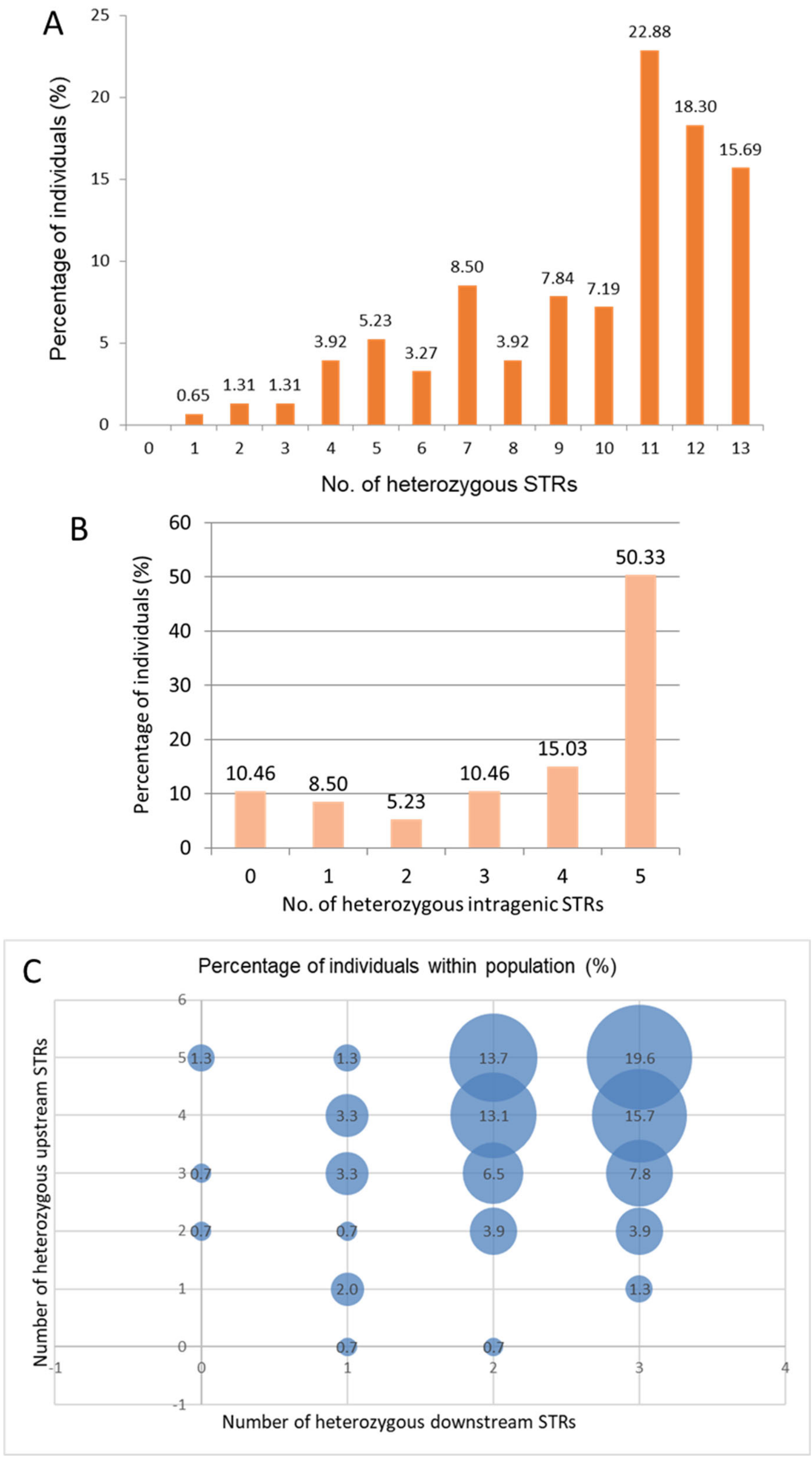

Supplemental Figure S1. Heterozygosities of *F8*-associated short tandem repeat (STR) markers in the Vietnamese Kinh population. (A) Distribution of individuals heterozygous for different numbers of STRs. (B) Distribution of individuals heterozygous for different numbers of intragenic STRs. (C) Distribution of individuals heterozygous for different numbers of STRs upstream and downstream of the *F8* gene.
